# A Case Report of Primary Pulmonary Lymphoepithelioma-Like Carcinoma with “Harmful” Pseudoprogression and a Pathological Complete Response (pCR) after Immunotherapy Plus Radiotherapy

**DOI:** 10.32604/or.2025.068300

**Published:** 2025-11-27

**Authors:** Si Qin, Shu Tang, Lijiao Xie, Jianbo Zhu, Jianguo Sun

**Affiliations:** Oncology Department, Xinqiao Hospital, Army Medical University, Chongqing, 400037, China

**Keywords:** Primary pulmonary lymphoepithelioma-like carcinoma, immunotherapy, radiation necrosis, pseudoprogression, case report

## Abstract

**Background:**

Primary pulmonary lymphoepithelioma-like carcinoma (PPLELC) is a rare subtype of primary non-small cell lung cancer (NSCLC), with no established treatment guidelines. We present a case of a young female with PPLELC who achieved a pathological complete response (pCR) in both primary and metastatic lesions after receiving combined immunotherapy and radiotherapy.

**Case description:**

We present a 33-year-old female patient with stage IVa (cT2bN0M1b) PPLELC. As a first-line treatment, the patient received seven cycles of nab-paclitaxel combined with toripalimab (a PD-1 inhibitor) and achieved stable disease. This was followed by toripalimab maintenance therapy for nearly 30 months. During toripalimab maintenance therapy, the patient demonstrated slight enlargement of both lung lesions and brain lesions. Radiotherapy was subsequently administered to both locations. However, after radiotherapy, the patient exhibited radiographic progression in both lesions with associated worsening of clinical symptoms. Surgical resection of the localized lesions was clinically warranted. Unexpectedly, the final postoperative pathology revealed a pCR. The patient maintained progression-free survival (PFS) exceeding 70 months, confirming that the prior radiographic progression represented pseudoprogression. Pseudoprogression is commonly defined as radiologic tumor progression from baseline that is not confirmed as progression on subsequent radiologic evaluation. Most of the patients experiencing pseudoprogression had a good performance status (PS), were paucisymptomatic, and even experienced the improvement of tumoral symptoms. In contrast, our case presented with worsening clinical symptoms and general conditions, which we term “harmful” pseudoprogression. To our knowledge, such a case of PPLELC with a “harmful” pseudoprogression is rarely reported; moreover, the term “harmful” pseudoprogression is our original creation.

**Conclusion:**

Our case highlights the critical role of re-biopsy and re-evaluation of imaging criteria in assessing the response to immunotherapy.

## 1 Introduction

Primary pulmonary lymphoepithelioma-like carcinoma (PPLELC) is a rare histological subtype of primary non-small cell lung cancer (NSCLC), accounting for approximately 0.7% of NSCLC [1]. Currently, due to the rarity of this cancer, no large-scale prospective trials have been conducted, and thus no established treatment protocols exist; most evidence to date relies on case reports and small-sample retrospective studies. PPLELC has been shown to be closely associated with Epstein-Barr virus (EBV) infection and shares clinical features with undifferentiated nasopharyngeal carcinoma (NPC) [2]. Most cases are from East Asia, and the majority occur in non-smokers (87.5%) and females (62.5%) [3]. Unlike other primary lung cancers, PPLELC has no obvious clinical manifestations and is often diagnosed at an advanced stage. It has been reported that nearly 50% of patients had stage IV disease at diagnosis [4]. Early-stage PPLELC patients are treated with curative resection and have a favorable prognosis, while late-stage patients have an unsatisfactory prognosis due to the lack of standardized treatment. Lin et al. reported median survival times of 54.1 months and 27.6 months for stage IIIA and IV PPLELC, respectively [5]. We herein present a case of advanced PPLELC that achieved pathological complete response (pCR) following combined therapy of radiotherapy and immunotherapy, with progression-free survival (PFS) exceeding 70 months. Interestingly, the patient developed pseudoprogression during the achievement of pCR. Unlike conventional pseudoprogression, this patient suffered significant aggravation of clinical symptoms and a notable decline in quality of life, which we term “harmful” pseudoprogression—A previously unreported clinical manifestation.

This study was approved by the ethics committee of Xinqiao Hospital, Army Medical University, with the approval number: 2025-231-01. The handwritten informed consent was obtained from the patient. Additionally, this study was prepared according to the CARE case report guidelines, with the corresponding checklist provided [6]. Please see the Supplementary Material for more details.

## 2 Case Presentation

**Figure 1:**
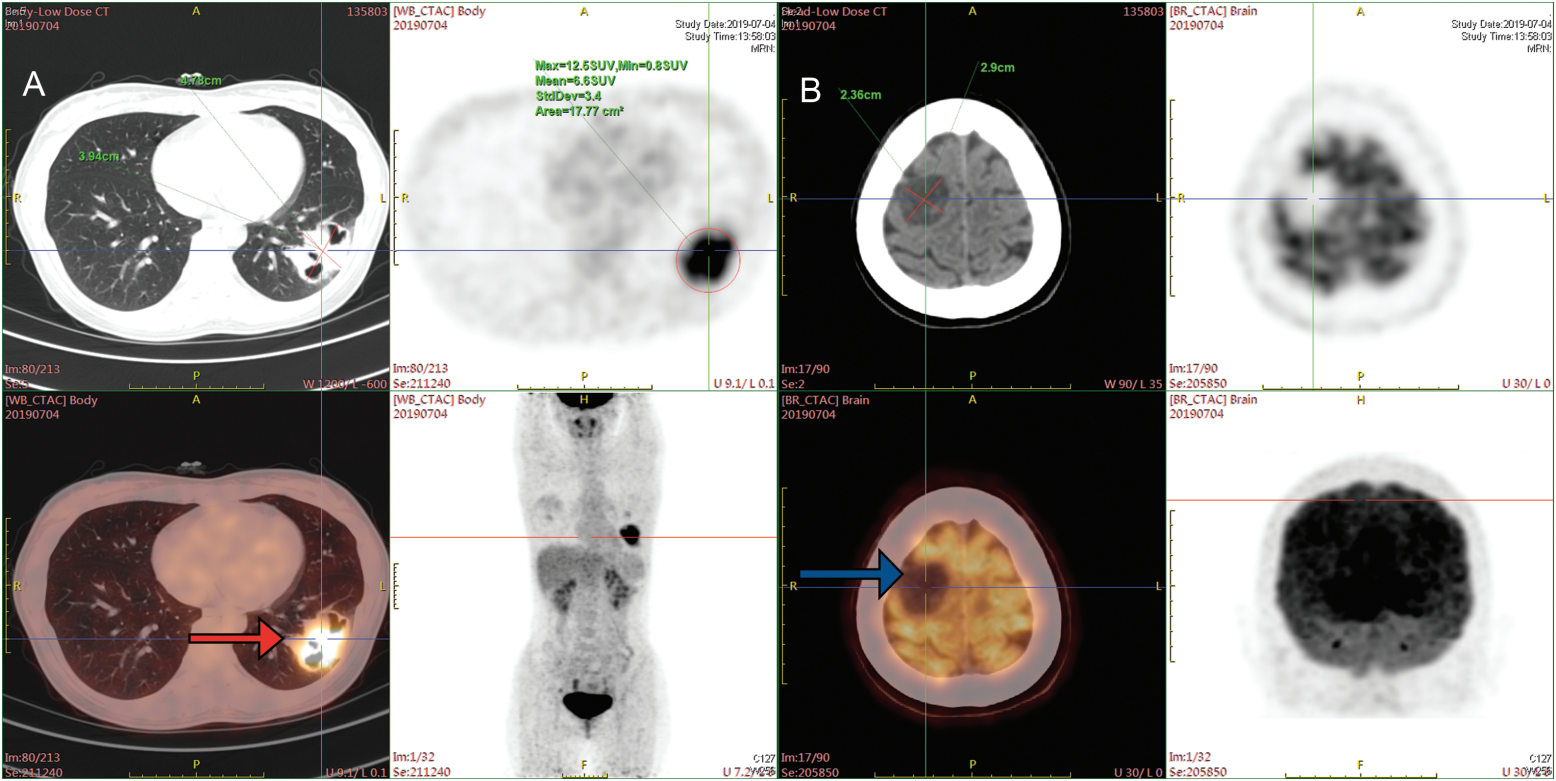
PET-CT images at the time of initial diagnosis. **(A)** PET-CT on 04 July 2019, before treatment. The mass at the peripheral basal segment of the left lower lobe showed increased FDG uptake (red arrow). **(B)** Low-density lesions in the right parietal lobe, with increased FDG metabolism (blue arrow). PET-CT = Positron emission tomography-computed tomography, FDG = fluorodeoxyglucose

A 33-year-old female presenting with a persistent cough and sputum containing dark red blood was admitted to Xinqiao Hospital of Army Medical University on 02 July 2019. She was a non-smoker and had no medical history. Upon admission, a physical examination was performed and revealed no significant abnormalities. Enhanced chest computed tomography (CT) revealed a mass in the lower lobe of the left lung, with a longest diameter of about 4.9 cm. Enhanced cranial magnetic resonance imaging (MRI) showed a tumor in the right parietal region, with a longest diameter of about 3.0 cm, which was considered suspicious for metastasis. Positron emission tomography-computed tomography (PET-CT) findings were consistent with these results (Fig. 1A,B). The patient underwent ultrasound-guided lung biopsy. The pathological result confirmed left lower lobe pulmonary lymphoepithelioma-like carcinoma, with immunohistochemistry showing P63+, P40+, Ki-67 50%, TTF-1-, NAPSINA-, SYN-, vimentin focal+. *In situ*, hybridization showed EBER+. No mutations or fusions were detected in EGFR, ROS1, RAS/RAF, or PIK3CA. Further examinations, including nasopharyngeal MRI and nasopharyngoscopy, showed no abnormalities. Based on the above examinations, the patient has been formally diagnosed with stage IVa lymphoepithelioma-like carcinoma of the left lower lung (cT2bN0M1b). We initiated an MDT discussion; at that time, both neurosurgeons and thoracic surgeons considered that this patient had no clear indication for surgery. In accordance with the NCCN guidelines and the patient’s wishes, she received seven cycles of nab-paclitaxel and toripalimab combination therapy, achieving stable disease (SD), followed by maintenance therapy with toripalimab.

**Figure 2:**
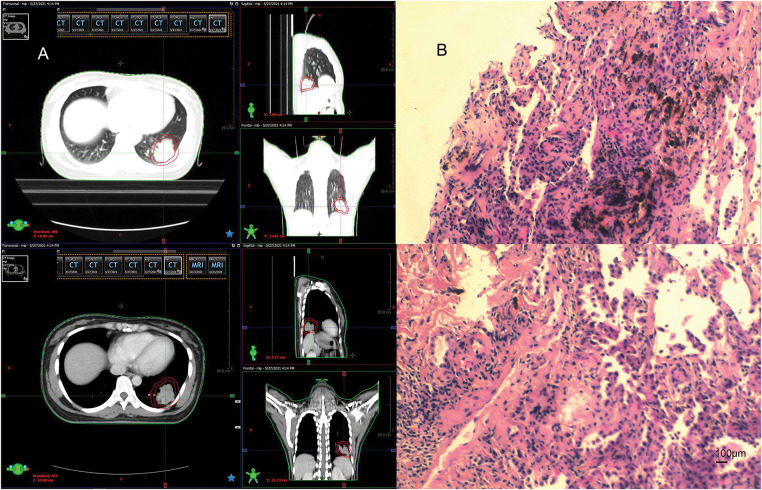
Radiotherapy plan and pathologic findings after lung lesion enlargement. (**A**) First stage radiotherapy of the left lung region. (**B**) Biopsy pathology of the lung lesion in December 2021 (left inferior lobe lesion). Chronic inflammation of puncture tissue, fibrous hyperplasia, and local deposition of char (original magnification × 100, scale bar = 100 μm)

**Figure 3:**
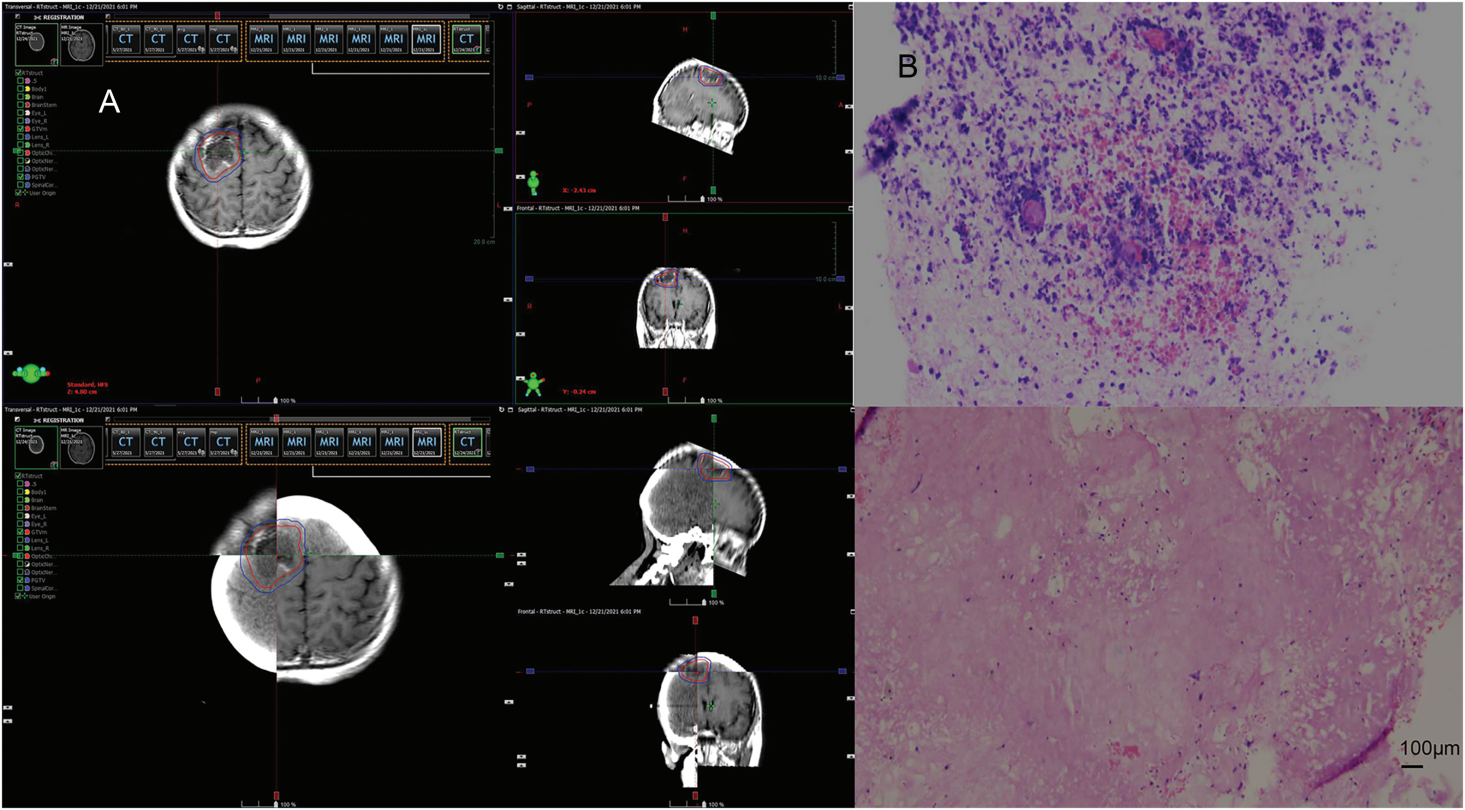
Radiotherapy plan and pathologic findings after brain lesion enlargement. (**A**) Second stage of the right parietal lobe region. **(B)** Postoperative pathology of the brain. Brain tissue edema with necrosis, focal neutrophil, and lymphocyte infiltration, mild glial cell proliferation, no definite neoplastic lesions (original magnification × 100, scale bar = 100 μm)

In April 2021, follow-up imaging of the chest enhanced computerized tomography revealed a slight enlargement of the pulmonary lesion; the rest of the systemic assessment was stable. Localized radiotherapy was initiated, with a treatment plan of intensity-modulated radiotherapy (IMRT) delivering a total dose of 64.4 Gray (Gy) in 14 fractions to the left lower lobe lesion (Fig. 2A). About one month after radiotherapy, the patient experienced a worsening cough and sputum production. In November 2021, a follow-up chest CT at a local hospital showed a significant enlargement of the left lower lobe lesion. The patient returned to our hospital for further examination. The findings were as follows: 1. A mass shadow in the lower lobe of the left lung, with a longest diameter of about 4.3 cm, is enlarged compared with prior imaging, FDG metabolism is slightly increased, post-treatment inflammatory changes are considered possible biopsy recommended if necessary; 2. right parietal lobe hypodense foci, FDG metabolism is reduced, the longest diameter of about 3.38 cm, slightly increased compared with the previous. A biopsy was performed again on the left lower lung lesion, and the pathologic result suggested chronic inflammation without evidence of cancer cells. Following departmental discussion, considering that surgery has both diagnostic and therapeutic functions, it was recommended that the left lower lung lesion be resected, the brain lesion be treated with radiotherapy, and immune maintenance therapy be discontinued. In January 2022, the patient underwent a radical resection of the left lower lobe of the lung, postoperative pathology (Fig. 2B): (left lower lung) chronic inflammation with histiocytic aggregation and multinucleated giant cell reaction, no residual cancer tissue, negative margin, lymph nodes (0/12). The cough symptoms of the patient were significantly relieved after the operation. Meanwhile, she completed radiotherapy for a brain lesion, with a total dose of 50 Gy in 10 fractions, targeting the probable gross tumor volume (PGTV) (Fig. 3A). One month after completion of brain radiotherapy, she developed right parietal headache and left-sided limb numbness, which worsened over time. In July 2022, the patient began to have convulsions in the left-sided limb, and the frequency gradually increased. In November 2022, the brain MRI showed that the right parietal lesion was revealed to be larger than before, with a longest diameter of about 4.0 cm. Referring to the management of lung lesions, the patient underwent a craniotomy for brain tumor resection in November 2022. The postoperative pathology (Fig. 3B): (right parietal lobe) cerebral edema with necrosis, neutrophilic and lymphocytic infiltration, mild proliferation of glial cells, and no definitive evidence of tumor. Similarly, the symptoms of headache and limb convulsion were significantly relieved after the operation.

In April 2023, the patient presented again with a cough and shortness of breath. The re-examination of the lung CT showed a new nodular hyperdense shadow in the operated area of the left lung, with recurrence not excluded. A sonography-guided biopsy of this lesion revealed chronic inflammation with lymphocytic and eosinophilic infiltration. Bronchoalveolar lavage fluid metagenomic next-generation sequencing (mNGS) testing indicated EBV infection. After treatment with valaciclovir antiviral and gamma globulin neutralizing antibody, the lung lesions were significantly reduced, and the symptoms of cough and shortness of breath were markedly improved. Therefore, the new lesion in the left lung was considered an inflammatory lesion.

**Figure 4:**
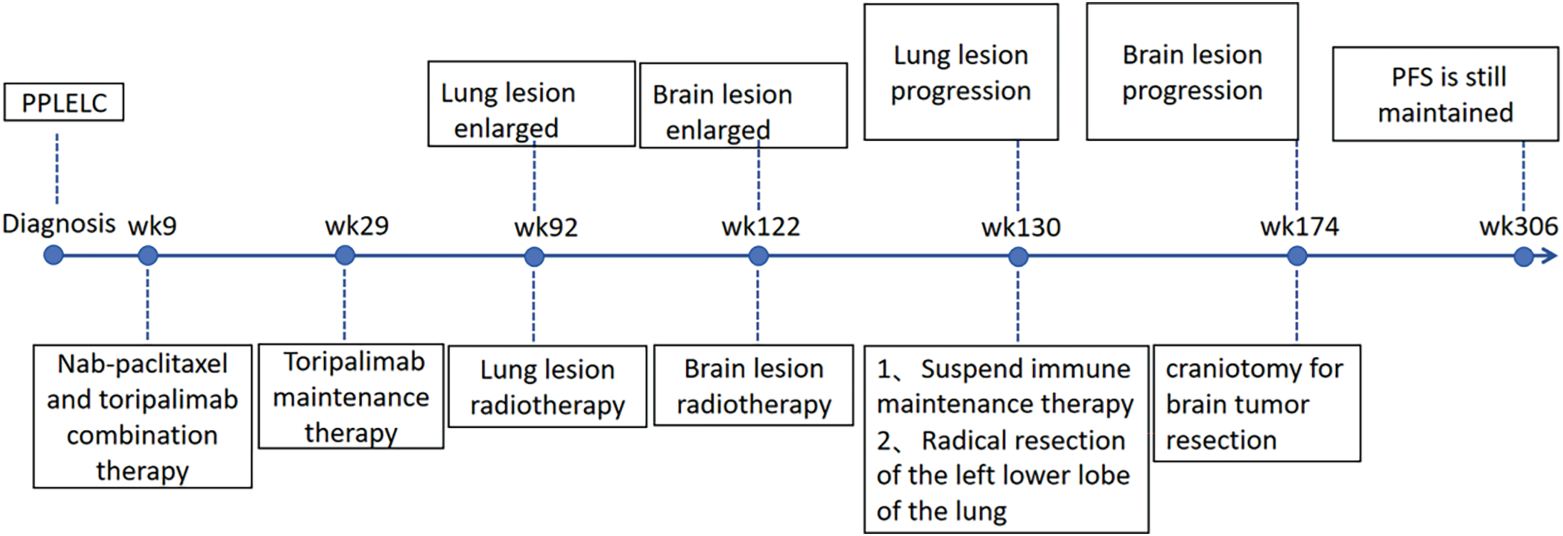
Timeline of the treatment

During regular follow-ups till May 2025, the patient has no clear evidence of tumor recurrence or metastasis, and the PFS is more than 70 months (Fig. 4).

## 3 Discussion

PPLELC is a poorly differentiated carcinoma characterized by dense lymphocytic infiltration in the stroma, which is associated with EBV infection [7]. EBV is a widespread tumor virus, especially endemic in East Asia, Southeast Asia, North Africa, and Polynesia [8]. Studies have shown that EBV-associated epithelial cancers, including Epstein-Barr virus-associated gastric cancers (EBVaGCs), nasopharyngeal carcinoma (NPC), and lymphoepithelial-like carcinoma (LELC), have in common a viral-mediated immune-suppressed tumor immune microenvironment, mutational signatures, and epigenetic hallmarks [9,10]. At present, how EBV contributes to PPLELC pathogenesis remains unclear. Png et al. proposed that PPLELC probably holds a similar model with NPC, whereby a type II latency program in dysplastic tissue, accompanied by other predisposing factors, leads to the development of cancer [11]. *In vitro* studies of nasopharyngeal carcinoma have shown that EBV has the ability to upregulate PD-L1 expression through IFN-γ and latent membrane protein 1 [12]. A meta-analysis showed that the incidence of programmed cell death-ligand 1 (PD-L1) expression in PPLELC was 63.3%–75.8% [13]. Chang et al. also noted that the proportion of PD-L1 expression in PPLELC was significantly higher than that in other NSCLCs, which further clarified that virus-associated tumor cells have dominant PD-L1 expression [14]. Therefore, the high expression of PD-L1 in PPLELC suggests the potential benefit of using immunotherapy in this subtype of lung cancer. Surgery is recommended for early-stage PPLELC, while the treatment strategy for advanced-stage PPLELC is referenced to that for NPC, including gemcitabine-based chemotherapy, taxanes, anti-angiogenic therapy, and anti-PD1/PD-L1 therapy [15].

PPLELC was reported to be sensitive to radiotherapy. Tang et al. suggested that radiotherapy could improve OS of PPLELC patients, which might be attributed to the similar biological characteristics with NPC [13]. Zuan Lin et al. evaluated the value of radiotherapy and concluded that palliative thoracic radiotherapy was beneficial for prolonging the survival of PPLELC patients with advanced-stage disease [16]. The immunotherapy and radiotherapy were considered to be a promising approach for improving the OS and cure rate of patients with resectable PPLELC lesions. We designed an individualized treatment strategy of immunotherapy plus radiotherapy for this patient. The patient achieved long-term PFS, which may be due to the massive necrosis of tumor cells after radiotherapy, the release of tumor antigens, the formation of vaccines *in situ*, and the complete activation of the immune system. The long-term CR of this patient must have a potential scientific basis. Regrettably, immunotherapy-related biomarkers such as PD-L1^+^, and CD4^+^, CD8^+^ T cell infiltration were not measured in this patient, which may enhance the efficacy of radiotherapy.

The advent of immune checkpoint inhibitors (ICIs) has significantly improved the survival rate of NSCLC patients, but a phenomenon known as pseudoprogression has been increasingly recognized [17]. Pseudoprogression, initially identified in temozolomide-treated brain malignancies, describes a transient increase in tumor size on imaging prior to therapeutic benefit [18]. This phenomenon was later recognized in melanoma patients receiving ICIs [19]. Pathologically, the observed radiographic enlargement corresponds to peritumoral immune cell influx (including cytotoxic T lymphocytes), edema, and necrotic changes, rather than genuine tumor growth [20]. Furthermore, delayed immune activation, potentially interacting with the inflammatory milieu of immune cell infiltration, is hypothesized to play a role in pseudoprogression, especially in cases where tumor shrinkage occurs after the pseudoprogressive event [21]. Conventional pseudoprogression, previous retrospective studies have shown that although it is accompanied by an initial increase of tumor size, it does not go with deterioration in clinical or performance status, such as weight loss, fever, night sweats, and increasing pain. In the case reported by Chae et al., although the pseudoprogression indicated a 163% increase from baseline tumor burden, the patient’s pain almost resolved, and fatigue disappeared with marked improvement in overall energy levels [22]. As for “harmful” pseudoprogression, it is often concomitant with the deterioration of the patient’s condition, very likely to be confused as true progress or even super-progress. We have created the concept of “harmful” pseudoprogression, the first is to highlight the deterioration of patients’ clinical symptoms, and the second is to emphasize that if it cannot be correctly identified, it is very likely to lead to incorrect subsequent treatment decisions. Most immunotherapy-induced pseudoprogression involves stable absorption after T cell enrichment [23]. Patients develop “harmful” pseudoprogression after radiotherapy, which can lead to inflammatory changes after T cell recruitment, leading to exacerbation of their symptoms. In our case report, the pseudoprogression caused by immunotherapy combined with radiotherapy is different from the progression of immunotherapy alone. This is a “harmful” pseudoprogression that causes further damage to the brain and lungs. We thought that “harmful” pseudoprogression could be benign nodules caused by EBV stimulation, which cause symptoms that look like disease progression due to the mass effect. It is recommended that all similar cases be followed up for further detection of EBV nucleic acids and antibodies, and EBER can also be included in pathological reports. If the disease is considered associated with EBV infection, anti-inflammatory, antiviral, and gamma neutralization antigen therapy can be given. In addition, we need to consider whether radiation necrosis is involved. In acute radiation-induced injury, transient vasodilatation occurs with variable changes in capillary permeability that sometimes manifest as vasogenic edema [24]. The pathological findings are mainly endothelial damage, vascular dilation, and telangiectasia. These changes have an effect on capillary permeability that produces cytotoxic and vasogenic edema [25]. One different concept is that cytokine release may promote angiogenesis, which is associated with capillary leakage. The main cytokine secreted after irradiation is tumor necrosis factor-alpha (TNF-α). TNF-α upregulates other cytokines that induce apoptosis of the endothelial cells, astrocyte activation, and blood-brain barrier (BBB) permeability. The vascular endothelial growth factor (VEGF) induces small vessel permeability and causes cerebral edema. VEGF expression has been associated with the magnitude of edema and breakdown of the BBB. The presence of radiation necrosis has been linked with increased VEGF expression [26].

Nevertheless, there are still many issues to be clarified. Although the patient’s postoperative pathology confirmed pCR, the patient’s preoperative CT and MRI evaluation still showed obvious lesions. This phenomenon might be attributed to radiotherapy’s activation of the immune system, leading to a significant influx of immune cells into the tumor tissue. Consequently, post-treatment CT and MRI images may demonstrate an increased tumor volume compared to pre-treatment scans. This situation prompts a critical question regarding the adequacy of current imaging modalities, such as CT and MRI, for assessing the effectiveness of immunotherapy treatments. It also raises the possibility that alternative imaging techniques, like PET-CT, could provide a more accurate evaluation. FDG-PET-CT is widely used in the screening for distant metastases and monitoring treatment response in patients treated with ICIs [27]. However, FDG is not a cancer-specific tracer. Immune system activation elicited by ICIs can lead to increased glycolytic use by the immune cells infiltrating the tumor, and thereby, increased FDG uptake is not only observed in the tumor cells but also in the tumor-infiltrating immune cells and within the tumor microenvironment. Therefore, there is a need to identify and develop additional tools and molecular imaging strategies that can differentiate metabolically active cancer cells from activated immune cells when evaluating tumor response in patients treated with ICIs. However, the physiological high FDG uptake in the normal brain parenchyma hinders the delineation of brain tumors, and cerebral inflammatory processes may also exhibit high FDG uptake, thereby diminishing the diagnostic performance [28]. Moreover, advanced MRI techniques, including perfusion-weighted imaging (PWI), MR spectroscopy (MRS), and diffusion-weighted imaging (DWI), offer valuable information concerning tumor biology, especially at the functional, physiologic, and molecular levels [29].

To our knowledge, this is the first reported case of PPLELC exhibiting “harmful” pseudoprogression following a combination of immunotherapy and radiotherapy. The patient was initially diagnosed with cranial metastasis and had no surgical indications. Consequently, treatment commenced with a combination of nab-paclitaxel and triprolizumab, followed by maintenance therapy with triprolizumab alone. Subsequent to an observed enlargement in tumor size, localized radiotherapy was administered to both the primary lung lesions and brain metastases. However, imaging examinations post-radiotherapy revealed an enlargement of both lung and brain lesions, accompanied by worsening clinical symptoms, leading to surgical intervention. Pathological examination of the resected lung lesion and brain metastasis ultimately showed a complete pathological response (pCR), with no residual tumor cells detected. Immunotherapy may be the preferred treatment for PPLELC, and combination therapy to improve the efficacy of immunotherapy (such as radiotherapy) may be the best choice. This case underscores the importance of recognizing pseudoprogression as a potential phenomenon in patients receiving combined immunotherapy and radiotherapy regimens, highlighting the need for careful monitoring and interpretation of imaging findings in assessing treatment response.

In summary, the introduction of “harmful” pseudoprogression highlights the critical importance of re-biopsy and re-evaluation of imaging criteria when assessing immunotherapy response. This case underscores the need for clinicians to vigilantly distinguish between true progression and pseudoprogression, particularly the “harmful” variant, which can significantly impair patients’ quality of life and is highly likely to be mistaken for true progression or even hyperprogressive disease (HPD). We hope this report provides new insights into the clinical management of PPLELC and contributes to refining treatment efficacy evaluation in similar cases.

## Supplementary Materials



## Data Availability

The datasets generated and/or analyzed during the current study are available from the corresponding author upon reasonable request.

## Supplementary Material

The supplementary material is available online at https://www.techscience.com/doi/10.32604/or.2025.068300/s1.
